# Association of congenital Zika syndrome with dental alterations in children with microcephaly

**DOI:** 10.1371/journal.pone.0276931

**Published:** 2022-11-01

**Authors:** Patrícia Nóbrega Gomes, Beatriz Aguiar do Amaral, Isabelita Duarte Azevedo, Haline Cunha de Medeiros Maia, Nivia Maria Rodrigues Arrais, Kenio Costa de Lima

**Affiliations:** 1 Department of Dentistry, Federal University of Rio Grande do Norte, Natal, Rio Grande do Norte, Brazil; 2 Department of Pediatrics, Federal University of Rio Grande do Norte, Natal, Rio Grande do Norte, Brazil; Ohio State University, UNITED STATES

## Abstract

The effects of congenital Zika syndrome (CZS) on the tooth development of infected children are not well known. The aim of this study was to analyze the association of CZS with dental alterations in children with microcephaly seen at a referral hospital in Rio Grande do Norte, Brazil. The chronology and sequence of tooth eruption and the presence of dental alterations were evaluated by a single calibrated examiner (kappa > 0.80) in 62 children aged 7 to 35 months with microcephaly associated with CZS and other congenital infections. Medical data of the mother and child were collected from the records and the parents responded to a socioeconomic questionnaire. Descriptive analysis and Fisher’s exact test were used (5% significance level). The mean age of the children was 26.4 months (SD = 7.52). The mean weight and head circumference at birth were 2,593 g (SD = 0.60) and 29.6 cm (SD = 2.48), respectively. Microcephaly was associated with congenital Zika virus infection in 79% of cases and with other congenital infections in 21%. No significant association was found between CZS and alterations in the chronology (p = 1.00) or sequence of tooth eruption (p = 0.16) or changes in enamel development (p = 1.00). In conclusion, children with microcephaly exhibit a delay and alterations in the sequence of tooth eruption of primary teeth, as well as developmental defects of enamel, which are not associated with Zika virus infection.

## Introduction

Zika virus is an arbovirus of the *Flavivirus* genus, which is transmitted by *Aedes aegypti* mosquitoes. The virus was first isolated in 1947 in the Zika forest located in Uganda [1]. The first known epidemic occurred in 2007 on the Yap island, Federal States of Micronesia, affecting 73% of the population [2]. The typical clinical presentation of Zika virus infection, when symptomatic, lasts 4 to 7 days. The main clinical manifestations include maculopapular rash, low fever, ocular hyperemia, arthralgia, myalgia, and headache [3–5].

In Brazil, cases with a clinical presentation compatible with Zika virus infection were reported at the end of 2014. In the beginning of 2015, the virus was isolated for the first time in the state of Bahia and from August of the same year, the Ministry of Health had identified an increase in the number of microcephaly cases among newborns, especially in the northeastern region of Brazil [6]. Investigations into its possible association with outbreaks of Zika virus infections began to occur [6, 7]. This association was confirmed in November 2015 by the detection of Zika virus-specific immunoglobulin M in cerebrospinal fluid of newborns with brain abnormalities, indicating the occurrence of congenital infection [8, 9].

Microcephaly is defined as a condition in which the head circumference (HC) is below the normal age- and sex-specific standard curves for children. New parameters to measure HC and to identify suspicious cases of infants with microcephaly were adopted by the Brazilian Ministry of Health in 2016, with a HC ≤ 31.9 cm for boys and ≤ 31.5 cm for girls. In the case of premature infants, the parameters of the InterGrowth table are used, which considers the gestational age of the child [10].

Other congenital infections such as syphilis (S), toxoplasmosis (T), rubella (R), cytomegalovirus (C), and herpes simplex (H), referred to by the acronym STORCH, can also cause microcephaly and other brain abnormalities in affected children [11, 12]. However, in addition to microcephaly, newborns exposed to Zika virus exhibit a series of changes in the growth and development, important neurological complications such as brain calcifications, hypertonia, craniofacial disproportion, spasms, seizures, and visual and auditive alterations that, together, comprise the so-called congenital Zika syndrome (CZS) [13–16].

The process of tooth development, or odontogenesis, begins around the sixth week of intrauterine life and is triggered by cells that migrate from the neural crest, the same embryonic tissue from which the central nervous system arises. Disturbances that occur during this period may lead to changes in the physiology and morphology of dental tissues, affecting their internal and external anatomy [17]. That would include maternal viral infections, as infection by Zika virus, affecting the development of the primary dentition [18].

The hypothesis of this study is that children with microcephaly due to congenital infections exhibit alterations in tooth development. Within this context, in view of the severe neurological manifestations induced by CZS, Zika virus infection may cause these changes to be more severe and more frequent. Therefore, this study aimed to analyze the association of CZS and other congenital infections with dental alterations in children with microcephaly.

## Materials and methods

This cross-sectional study was conducted in the pediatrics outpatient clinic of the Onofre Lopes University Hospital (HUOL), Federal University of Rio Grande do Norte (UFRN), Brazil, between May 2018 and March 2019. The study was approved by the Research Ethics Committee of UFRN (Approval No. 1.717.592). The written informed consent was obtained from all participants for inclusion in the study.

The sample consisted of all children with microcephaly associated with congenital Zika virus infection and other congenital infections seen by a multiprofessional project of HUOL, a referral center for these cases in Rio Grande do Norte – Brazil, totaling 62 children.

After the parents/legal representative of all participants included in the study had signed the free informed consent form, a single researcher performed the interviews and clinical examinations. The children were submitted to oral clinical examination in the knee-to-knee position. Individual and sterile flat mouth mirrors No. 3 (Golgran^®^), sterile gauze, and a headlamp (CREE T6 – VT-169, DP^®^) were used for clinical examination. Data regarding the chronology and sequence of tooth eruption, presence of dental alterations (number, size, and type), and developmental defects of enamel (DDE) were collected.

The chronology and sequence of eruption of primary teeth were evaluated using the table proposed by Logan and Kronfield [19] and modified by Lunt and Law [20]. Developmental defects of the enamel were analyzed using the modified developmental defects of enamel (DDE) index, which classifies them into opacities and hypoplasia [21]. Intraexaminer calibration was performed by reevaluating 10% of the sample with one week interval. Agreement between pairs was obtained using the kappa coefficient, which was higher than 0.80.

Demographic data and socioeconomic conditions were collected from the questionnaire completed by the parents/legal representative. Information about mother’s pregnancy and birth of the child were obtained from the hospital medical records as was the type of child’s congenital infection, the diagnosis of which followed the criteria of the Ministry of Health of Brazil for the classification of congenital infections. This classification includes the presence of two or more of the main findings related to CZS, the presence or absence of reports of fever or exanthema without a defined cause during pregnancy, and the laboratory results for Zika and other congenital infections in maternal and newborn samples [14].

### Statistical analysis

The data were analyzed using the Statistical Package for the Social Sciences (SPSS® for Windows, version 24; SPSS, Inc., Chicago, IL, USA). Descriptive analysis was used and associations were evaluated by the chi-square test and Fisher’s exact test, adopting a level of significance of 5%.

## Results

The sample of this study consisted of 62 children with a mean age of 26.4 months (SD = 7.52). There were 32 (51.6%) boys and 30 (48.4%) girls. Table 1 shows the socioeconomic characteristics of the children.

**Table 1:** Socioeconomic characteristics of children with microcephaly.

Socioeconomic variables	*n*	%
**Living with**		
Mother and father	47	75.8
Other situations	15	24.2
**Property owned**		
Yes	06	12.8
No	35	76.1
**Working mother**		
Yes	6	9.7
No	56	90.3
**Maternal education level**		
Up to 9 years of schooling	31	50
> 9 years of schooling	31	50
**Monthly household income**		
Up to 2 minimum wages	46	74.2
> 2 minimum wages	16	25.8
**Household size**		
Up to 2 persons/room	42	67.7
> 2 persons/room	20	32.3

MW: minimum wage (R$ 954,00)

Regarding the children’s birth data, the mean gestational age was 37.9 weeks (SD = 2.48). The mean weight and HC at birth were 2,593 g (SD = 0.60) (11,65 in) and 29.6 cm (SD = 2.48) (0,005 lbs), respectively. The mean Apgar score was 7.45 (SD = 1.48) in the first minute and 9.0 (SD = 1.61) in the fifth minute.

Microcephaly was associated with congenital Zika virus infection (CZS) in 79% (*n* = 49) of the cases and with other congenital infections in 21% (*n* = 13). The latter included congenital infection with STORCH in five cases (4 with cytomegalovirus and 1 with toxoplasmosis) and congenital infection without etiological identification but with negative serology for Zika virus in eight cases.

Table 2 shows the results of comparative analysis of the independent variables according to the presence and absence of alterations in the chronology and sequence of tooth eruption.

**Table 2:** Comparative analysis of independent variables according to the presence and absence of alterations in the chronology and sequence of tooth eruption.

	Altered chronology of tooth eruption				Altered sequence of tooth eruption			
	Yes *n* (%)	No *n* (%)	p-value	PR (95% CI)	Yes *n* (%)	No *n* (%)	p-value	PR (95% CI)
**Prematurity**								
Yes	19 (100)	0 (0.0)	0.546	------------	13 (72.2)	5 (27.8)	0.628	0.73 (0.20-2.60)
No	40 (93.0)	3 (7.0)			32 (78.0)	9 (22.0)		
**Low birthweight**								
Yes	30 (96.8)	1 (3.2)	1.000	2.06 (0.17-24.07)	22 (73.3)	8 (26.7)	0.590	0.71 (0.21-2.40)
No	29 (93.5)	2 (6.5)			23 (79.3)	6 (20.7)		
**Microcephaly**								
CZS	46 (93.9)	3 (6.1)	1.000	------------	33 (71.7)	13 (28.3)	0.159	0.21 (0.25-1.79)
Other congenital infections	13 (100)	0 (0.0)			12 (92.3)	1 (7.7)		
**Maternal education level**								
Up to 9 years	30 (96.8)	1 (3.2)	1.000	2.06 (0.17-24.07)	20 (69.0)	9 (31.0)	0.195	0.44 (0.12-1.53)
> 9 years	29 (93.5)	2 (6.5)			25 (83.3)	5 (16.7)		
**Monthly income**								
≤ 2 MW	43 (93.5)	3 (6.5)	0.562	------------	31 (70.5)	13 (29.5)	0.090	0.17 (0.20-1.43)
> 2 MW	16 (100)	0 (0.0)			14 (93.3)	1 (6.7)		

CZS: congenital Zika syndrome; MW: minimum wage (R$ 954,00); PR: prevalence ratio; CI: confidence interval.

Children with microcephaly had a high frequency of alterations in the chronology and sequence of tooth eruption. However, no statistically significant association was observed between congenital Zika virus infection and these alterations.

Comparative analysis of the independent variables according to the presence and absence of DDE is shown in Table 3.

**Table 3:** Comparative analysis of independent variables according to the presence and absence of developmental defects of enamel.

	Developmental defects of enamel			
	Yes *n* (%)	No *n* (%)	p-value	PR (95% CI)
**Prematurity**				
Yes	15 (83.3)	3 (16.7)	0.516	1.83 (0.44-7.57)
No	30 (73.2)	11 (26.8)		
**Low birthweight**				
Yes	24 (80.0)	6 (20.0)	0.493	1.52 (0.45-5.10)
No	21 (72.4)	8 (27.6)		
**Microcephaly**				
CZS	35 (76.1)	11 (23.9)	1.000	0.95 (0.22-4.09)
Other congenital infections	10 (76.9)	3 (23.1)		
**Maternal education level**				
Up to 9 years	23 (79.3)	6 (20.7)	0.590	1.39 (0.41-4.67)
> 9 years	22 (73.3)	8 (26.7)		
**Monthly income**				
≤ 2 MW	34 (77.3)	10 (22.7)	0.738	1.23 (0.32-4.74)
> 2 MW	11 (73.3)	4 (26.7)		

CZS: congenital Zika syndrome; MW: minimum wage (R$ 954,00); PR: prevalence ratio; CI: confidence interval.

There was also no significant association between congenital Zika virus infection and the presence of DDE in the children examined. Opacity was the most common developmental defect (71%). Enamel hypoplasia was observed in only 5% (*n* = 3) of the sample.

Prematurity, low birthweight and socioeconomic variables were not significantly associated with any of the dental alterations evaluated. Taken together, the dental alterations of number, shape and size represented 6.2% of the sample.

## Discussion

During pregnancy some infectious diseases can be transmitted to the fetus, commonly through the transplacental hematogenous route. The most common intrauterine infections are those grouped under the STORCH acronym – syphilis, toxoplasmosis, rubella, cytomegalovirus, and herpes simplex [11, 12]. After the Zika virus epidemic that occurred from August of 2015 and its association with an increase in the number of reported microcephaly cases in Brazil, this pathogen was added to the STORCH acronym, which became STORCH + ZIKA [14]. These infections in pregnancy can have severe consequences for the health and development of the fetus [11–13].

Regarding dental alterations, this study showed a high frequency of alterations in the chronology and sequence of eruption of primary teeth in children with microcephaly. However, no significant difference in proportions was observed between Zika virus infection and other congenital infections. Previous studies also reported a delay in the eruption of primary teeth and alterations in the sequence of eruption in children with CZS-related microcephaly [22–28].

Several factors can influence the process of tooth eruption, including prematurity, low birthweight, socioeconomic level, nutritional status, and infant eating habits [29–32]. However, there was no significant association between socioeconomic variables, prematurity or low birthweight and altered tooth eruption. This finding can probably be explained by the small size of the sample.

The high frequency of alterations in the chronology and sequence of tooth eruption observed in this study might be related to the type of food consumed by the children. Due to the presence of oropharyngeal dysphagia, a common condition in children with microcephaly [33], the foods consumed are less consistent and this can influence the pattern of dental occlusion [24].

The presence of DDE in children with congenital infections has been reported in previous studies [22, 23, 26, 28]. Furthermore, a history of infectious and congenital diseases such as congenital syphilis and rubella has been suggested as a factor predisposing to DDE in the primary and permanent dentition [34]. According to Jaskoll et al. [35], more than one-third of children with congenital cytomegalovirus infection born each year in the United States have enamel hypoplasia and opacity.

A 2018 study reported a high prevalence of enamel opacities in children with microcephaly associated with CZS [22]. In both groups in the present study the frequency of DDE was high (> 70%) indicating that these children are at an increased risk of developing caries and tooth sensitivity [36, 37].

Limitations of this study included the small sample size, the small number of microcephaly cases associated with other congenital infections and the short reevaluate interval in the intraexaminer calibration.

Contrary to the initial hypothesis, this study found no significant association between the groups and the frequency of dental alterations suggesting that the presence of microcephaly and the neurological damage resulting from it may represent risk factors for these alterations and not necessarily the infection with Zika virus.

## Conclusions

Children with microcephaly exhibit a delay and alterations in the sequence of tooth eruption and DDE in primary teeth. Congenital infection with Zika virus, which may result in microcephaly, was not associated with the occurrence of dental alterations.

## Supporting information

S1 Data (XLSX)
